# Spatial analysis of polarimetric images to enhance near-surface sampling sensitivity: feasibility in demineralized teeth and other tissue-like media

**DOI:** 10.1117/1.JBO.28.10.102906

**Published:** 2023-09-09

**Authors:** Michael D. Singh, Lothar Lilge, Alex Vitkin

**Affiliations:** a University of Toronto, Department of Medical Biophysics, Temerty Faculty of Medicine, Toronto, Ontario, Canada; b University Health Network, Princess Margaret Cancer Centre, Toronto, Ontario, Canada; c University of Toronto, Temerty Faculty of Medicine, Department of Radiation Oncology, Toronto, Ontario, Canada

**Keywords:** polarimetry, linear and circular polarization, penetration/sampling depth, biomedical optics, dental caries, Monte Carlo

## Abstract

**Significance:**

Early tooth demineralization may be detectable through spatial analysis of polarized light images as demonstrated in this study. This may also prove useful in the early detection of epithelial tumors that comprise the majority of the cancer burden worldwide.

**Aim:**

The spatial properties of polarized light images have not been greatly exploited in biomedicine to improve sensitivity to superficial tissue regions; therefore, we investigate the optical sampling depth effects as a function of location in the backscattered polarimetric images.

**Approach:**

Backscattered linear polarization intensity distributions exhibit four-lobed patterns arising through single-scattering, multiple-scattering, and geometrical effects. These photon pathway dynamics are investigated through experimental imaging of microsphere suspensions along with corroborative computational polarization-sensitive Monte Carlo modeling. The studied sampling depth effects of linear and circular polarization images (explored in a previous study) are then evaluated on normal and demineralized human teeth, which are known to differ in their surface and sub-surface structures.

**Results:**

Backscattered linear polarization images exhibit enhanced sensitivity to near-surface properties of media (for example, surface roughness and turbidity) at specific locations within the four-lobed patterns. This yields improved differentiation of two tooth types when spatially selecting image regions in the direction perpendicular to the incident linear polarization vector. Circular polarimetric imaging also yields improved differentiation through spatial selection of regions close to the site of illumination. Improved sensitivity to superficial tissues is achieved through a combination of these linear and circular polarimetric imaging approaches.

**Conclusions:**

Heightened sampling sensitivity to tissue microstructure in the surface/near-surface region of turbid tissue-like media and dental tissue is achieved through a judicious spatial selection of specific regions in the resultant co-linear and cross-circular backscattered polarimetric images.

## 1 Introduction

Polarization gating has been used in biophotonics to probe superficial tissue regions by filtering away deeply penetrating depolarized light.^1^ Notable early demonstrations of this include the works of Anderson et al.^2^ and Jacques et al.,^3^^,^^4^ which employed linear co- and crossed-polarization configurations to enhance visualization of dermatologic features and pathologies. The Feld group^5^^–^^7^ applied this type of filtering to optimize measurements of cell nuclear size in epithelial tissue for cancer detection applications. These “simple” polarization-filtering methods have been advanced in combination with other modalities, such as coherence-gating,^8^^,^^9^ temporal-gating,^10^^–^^12^ depolarization,^13^^–^^17^ and illumination property considerations (e.g., intensity, angle, and wavelength),^18^^–^^22^ to gain greater control over sampling depths and enable characterization of superficial tissues and pathologies. Yet it seems as if polarization-sensitive light collection properties (geometry, detector size, ROI selection, etc.)^18^ have thus far not been greatly exploited to enhance depth selectivity.

**Fig. 1:**
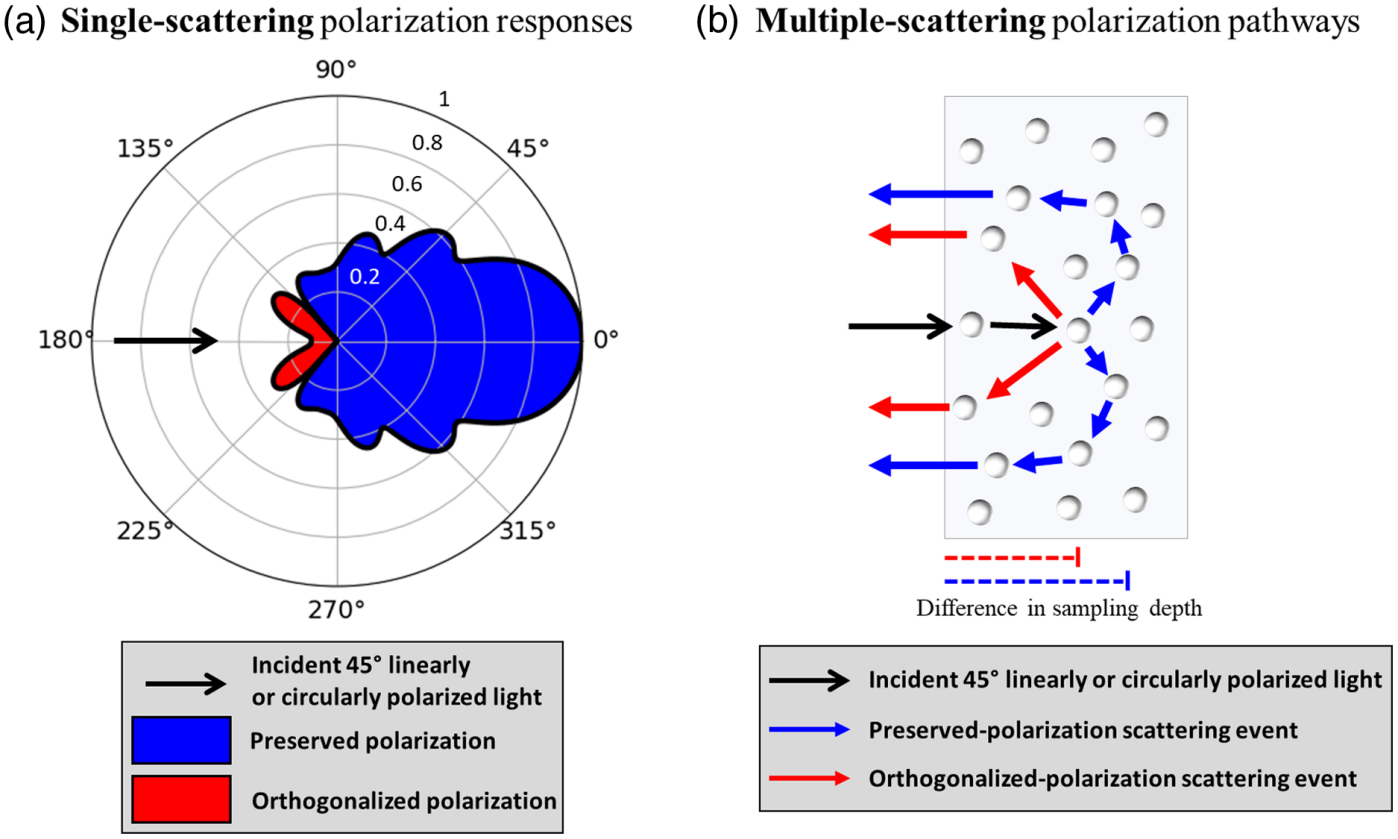
Polarization-dependent single-scattering and multiple-scattering effects. (a) Polar plot showing the scattered 45 deg linear and circular polarization intensities from incident 45 deg linearly and circularly polarized light, respectively, and color-coded red/blue to indicate orthogonalization/preservation. It is observed that light is orthogonalized in the backward-scatter direction (135 deg to 225 deg). (b) The single-scattering responses in (a) suggest that orthogonalized and polarization-state-preserving photons undergo characteristic multiple-scattering pathways in turbid media, such that the former penetrate less deeply. Figures adapted from Ref. 23.

Our previous study^23^ showed that useful depth sensitivity can be achieved through judicious selection of specific regions within backscattered circular co- and crossed-polarization images (i.e., helicity-preserved and helicity-flipped images, respectively). Specifically, helicity-flipping typically occurs after reflection-like large-angle backward-scattering events, whereas helicity-preservation occurs after small-angle forward-scattering events [see Fig. 1(a)]. Due to these scattering-angle-dependent responses, helicity-flipped light offers shallower sampling since it can be redirected into the backward hemisphere through a single reflection-like event, whereas helicity-preserved photons must be forward-scattered in an arc-like trajectory, which generally results in deeper penetration [see Fig. 1(b)].^24^^–^^26^ The superficial sensitivity of helicity-flipped light can then be further enhanced by spatially selecting the region that is closer to the illuminated spot since penetration depth generally increases with radial distance.

Notably, linearly polarized backscattered light can also exhibit co- and cross- spatial behavior with corresponding sensitivity to sampling depth,^25^^,^^27^ although arguably in a more complex fashion. Namely, four-lobed patterns emerge in co- and cross-linear images, such as those observed in early polarized light images of the human eye,^28^^–^^30^ atmospheric clouds,^31^^,^^32^ and other media, which have generated much curiosity (originally termed “polarization cross” patterns). These patterns are a result of interplays between single- and multiple-scattering effects of linearly polarized light. The sampling depths along certain directions of these patterns evidently offer shallower sampling depths. Though these patterns have been investigated,^33^^–^^36^ to the best of our knowledge they have not been exploited for their photon pathlengths/depth sensitivity characteristics.

To extend our previous work on circular polarization and depth sampling,^23^ here we investigate backscattered linear polarization patterns to gain insight into their sampling depth distributions. This is achieved through experimental imaging of polystyrene microspheres along with theoretical analysis, including single-scattering mathematics and corroborative Monte Carlo simulation statistics (e.g., intensity and scattering count distributions). It is demonstrated that superficial sensitivity to media can be enhanced through specific region selection of co-linear polarimetric images. Coupled with our earlier investigation of backscattered circularly polarized light patterns,^23^ a powerful composite polarimetry platform for differential depth sensitivity in an otherwise depth-unresolved technique emerges.

To assess the utility of this approach, we then applied it to differentiate normal and decayed human teeth, which exhibit different surface/sub-surface structural and compositional properties. It is found that optimal differentiation is achieved through specific spatial selection of polarimetric image regions using both linearly and circularly polarized light. The encouraging results may prove useful for the detection of early tooth decay, an important pre-requisite for effective intervention.^37^^,^^38^ Early tooth decay detection remains challenging due to the shallowness of demineralization in its early stages,^39^ highlighting the potential of our polarimetric method; however, future studies on larger sample sizes are needed. Nonetheless, successful differentiation suggests feasibility in real tissue milieu and reaffirms the findings of the tissue phantom study. Further methodology improvements, for example, via angularly resolved measurements,^40^ are envisioned.

## 2 Methods

### 2.1 Experimental Polarimetric System

The configuration and detailed description of the polarimetric system can be found in Ref. 23. Briefly, this setup is designed to generate circularly and linearly polarized light and detect the linear and circular co- and crossed-polarized components in the exact (180 deg) backscattering direction. This was done using a linear polarizer (P1) and quarter-wave retarder (R1) in the illumination axis, and linear analyzer (P2) followed by a quarter-wave retarder (R2) in the detection axis. Samples included microsphere suspensions and teeth, as described below. The light source for the suspensions was a continuous-wave laser diode operating at λ=635 nm. The light source for the teeth was a continuous-wave helium–neon laser operating at λ=543.5  nm. The beam diameter of the light sources was ∼3  mm (full width at half-maximum), with a ∼Gaussian intensity profile. The detection device was an intensified charge-coupled device (ICCD) camera (PI-MAX^®^ 3, Princeton Instruments), which output 1024×1024  pixel images, though only the central 400×400  pixel region was used for the results reported below. The microsphere suspensions were contained in a plastic cuvette of length 2.2 cm and imaged via a 2.2×2.2  cm silica optical window. The cuvette was angled slightly off-axis to avoid specular reflection from the air–glass and glass–water interfaces. The teeth were placed in upright positions and the crowns of each tooth were illuminated. Unlike the cuvette, the teeth were angled for maximal detection of specular reflection to enhance optical sensitivity to their surfaces.

### 2.2 Microsphere Suspensions

Monodispersed polystyrene microspheres (Bangs Laboratories, Inc.) of d=1.04  μm diameter were suspended in deionized water to yield a scattering coefficient of $\mu_{s}=25\;{\mathrm{cm}}^{-1}$, which is approximately the scattering coefficient of the enamel layer of human teeth at λ≈543.5  nm.^41^ The spheres had a refractive index of n=1.59 and the host medium (deionized water) had a refractive index of n=1.33.

### 2.3 Tooth Samples and Preparation

Teeth were provided by Dr. Anil Kishen from the University of Toronto School of Dentistry, following standard extraction. Upon visual inspection teeth were considered healthy with an intact enamel surface. Teeth were stored for up to 1 month in ethanol prior to use. Tested teeth included incisors and molars. For artificial induction of demineralization, teeth were oriented “sideways” and submerged halfway in 37% phosphoric acid (i.e., the biting surface was perpendicular to the acid interface; see the acidification line in Fig. 5). Phosphoric acid at this concentration is commonly used to induce demineralization and emulate natural tooth decay.^42^ Seven teeth were studied. Four measurements were taken on each tooth: two on the normal side and two on the acidified side. In total, there were thus 28 data points. Each tooth was submerged for specific times: (tooth 1: 1 min, t2: 5 min, t3: 10 min, t4: 25 min, t5/6/7: 40 min).

### 2.4 Monte Carlo Simulation Platform

The simulation parameters were configured to match the phantom experiment: $∼10^{8}$ polarized photon packets (λ=635  nm) were launched into a 2.2×2.2×2.2  cm medium of monodispersed 1.04  μm spheres, each with a refractive index of 1.59, suspended in a host refractive index of 1.33. Remaining details and description of the experimentally validated and publicly available Monte Carlo simulation platform^23^^,^^43^^–^^47^ can be found in Ref. 48 and Sec. S1.2 in Ref. 23.

## 3 Theoretical and Experimental Spatial Analysis of Polarimetric Images: Tissue Phantoms

To gain insight into the polarization-dependent backscattering dynamics, we first study single scattering, then connect those concepts to multiple scattering pathways through experimental imaging, corroborated and supplemented by computational modeling-based statistics. To do so, a polystyrene microsphere suspension is employed to greatly simplify the scattering dynamics through its narrow size distribution and discrete bi-phase refractive index profile. This enables convenient simulations of polarized light propagation, for example, as done in our recent studies.^23^^,^^47^

The angular distribution of singly scattered polarized light can be calculated by multiplying the Mueller matrix of a sphere with a given Stokes vector:

$$S_{\text{out}}=M_{s}\times S_{\text{in}}$$ (1)

$$[\begin{array}{c} I_{\text{out}}\\ Q_{\text{out}}\\ U_{\text{out}}\\ V_{\text{out}} \end{array}]=[\begin{array}{cccc} M_{11}(\theta ) & M_{12}(\theta ) & 0 & 0\\ M_{12}(\theta ) & M_{11}(\theta ) & 0 & 0\\ 0 & 0 & {\;M}_{33}(\theta ) & M_{34}(\theta )\\ 0 & 0 & -M_{34}(\theta ) & M_{33}(\theta ) \end{array}]\times [\begin{array}{c} I_{\text{in}}\\ Q_{\text{in}}\\ U_{\text{in}}\\ V_{\text{in}} \end{array}],$$ (2)

$M_{s}$ represents the Mueller matrix of a single scatterer. The 16 matrix elements can be determined through a Mie scattering calculation; the reader is referred to rigorous textbook summary by Bohren and Huffman^49^ for details. The values of the 16 elements also depend on the scattering angle θ, the angle between the incident and scattered light vectors (note that the detection plane is kept at a constant azimuthal angle of 0 deg for these calculations). $S_{\text{out}}$ and $S_{\text{in}}$ are the scattered and incident Stokes vectors, respectively.

Using Eq. (2), the scattered polarization intensity from a given incident polarization state can be calculated. For example, incident right-circularly polarized $S_{\text{in}}={\left[1,0,0,1\right]}^{T}$ yields the scattered circular polarization intensity of $V_{\text{out}}=\pm M_{33}$, where the negative sign indicates orthogonalization (i.e., a helicity-flip such that incident right-circular leaves as left-circular polarization). Interestingly, the scattered 45 deg linear polarization from incident +45  deg linearly polarized light, $S_{\text{in}}={\left[1,0,1,0\right]}^{T}$, is analogous (equivalent) to the circular case above, expressed as $U_{\text{out}}=\pm M_{33}$. Again, the negative sign indicates orthogonalization of the polarization vector (from +45  deg to −45  deg orientation). This equivalency is important conceptually and advantageous technologically in helping measure and interpret the two separate signal channels that detect polarization orthogonalization/preservation.

The scattered circular ($V_{\text{out}}$) and 45 deg linear ($U_{\text{out}}$) polarization intensities from a single polystyrene sphere are theoretically calculated as a function of scattering angle (assuming azimuthal symmetry) and plotted on a polar graph in Fig. 1(a) using Mie parameters λ=635  nm, host refractive index $n_{m}=1.33$ (water), and scatterer refractive index $n_{s}=1.59$ (polystyrene). It is observed that orthogonalization occurs in the backward direction between 135 deg and 225 deg (exact backscattering ±45  deg cone). This angular dependency suggests that linearly orthogonalized photons will exhibit shallower pathways in a polystyrene microsphere suspension (on average), as already shown previously for circularly polarized light.^23^ The orthogonalized and preserved (arc-like) pathways are schematized in Fig. 1(b) for incident circularly polarized and 45 deg linearly polarized light to conceptualize the difference in sampling depth. Thus, detection of orthogonalized photons likely enhances sensitivity to reduced sampling depths. Furthermore, due to the equivalency between $U_{\text{out}}$ and $V_{\text{out}}$, they can be used in combination to exploit this sampling depth selectivity effect.

Thus, to maximize superficial-region sensitivity via orthogonalized circular-polarization photons in the backward hemisphere, (1) a cross-circular setup can be employed (which detects helicity-flipped light) and (2) light that is closer to the region of illumination must be selected, as previously shown in fig. 6 in Ref. 23. Indeed, this approach led to an ∼20% reduction in average pathlength.^23^ The latter is important since helicity-flipped photons that exist in the radially distant regions arise through randomization/depolarization, which can be deeply penetrating. Circular polarization imaging and associated sampling depth effects were previously explored in detail in Ref. 23; here, we thus focus on linear polarization dynamics and associated image analysis.

**Fig. 2:**
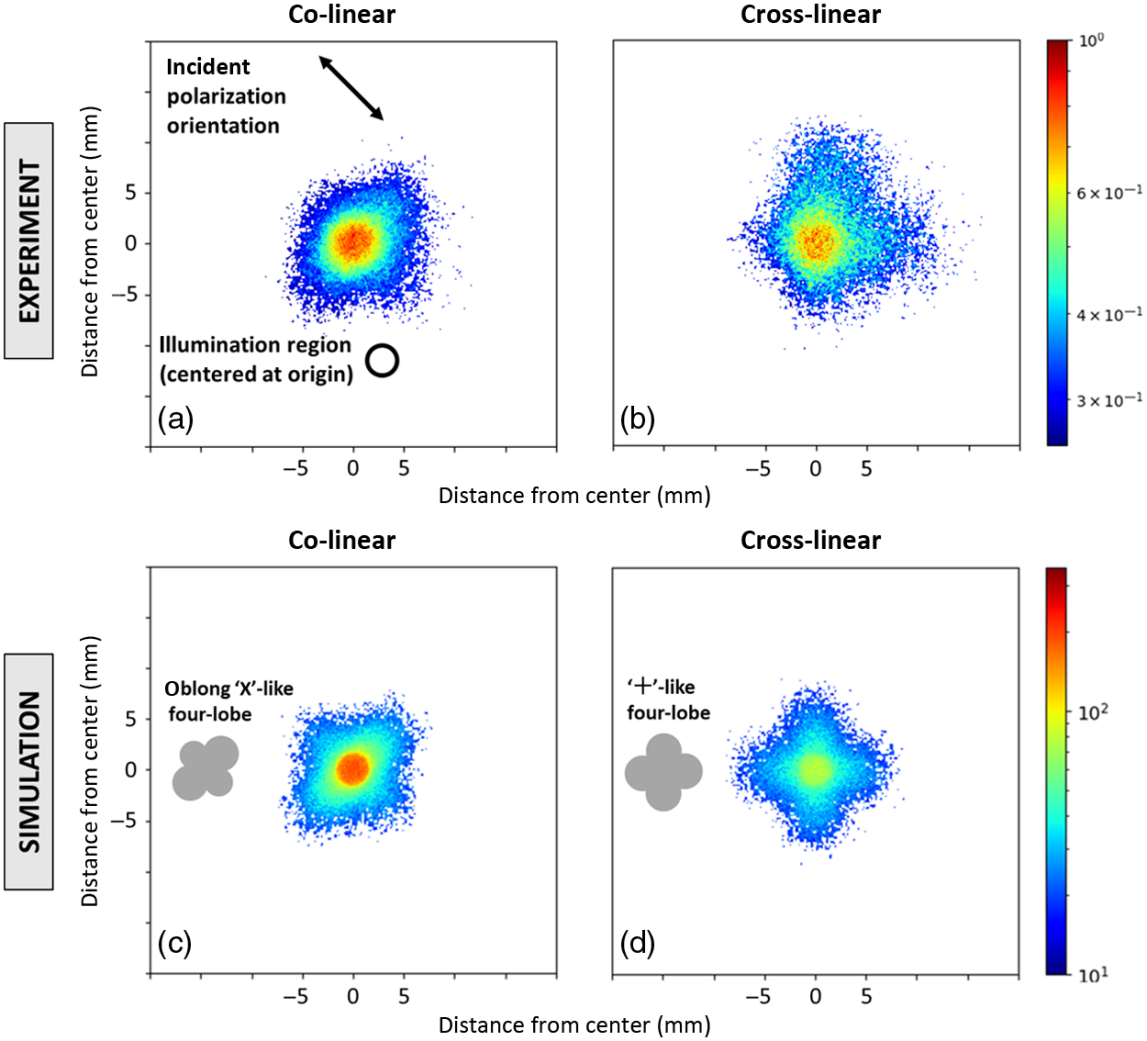
(a), (b) Experimental and (c), (d) simulated co-linear and cross-linear images of backscattered linearly polarized light from a 1.04  μm polystyrene microsphere suspension of $\mu_{s}=25\;{\mathrm{cm}}^{-1}$ turbidity. Co-linear images take on an oblong “×”-like four-lobed pattern, whereas cross-linear images take on a “+”-like four-lobed pattern. These patterns are formed through geometrical effects due to multiple scattering (see text for details).

Co- and cross-images of backscattered linearly polarized light from the polystyrene microsphere suspension were obtained experimentally [Figs. 2(a) and 2(b)] and through Monte Carlo computational modeling [Figs. 2(c) and 2(d)]. The microsphere suspension was illuminated at the origin by −45  deg linearly polarized light [incident beam size indicated in Fig. 2(a)]. We will focus our analysis on the simulation images since they are less noisy (easier to analyze) and agree well with the experimental results. Both the co- and cross-linear images exhibit “four-lobed” patterns.^34^^,^^36^ The co-linear image exhibits lobes along the axes that are parallel and perpendicular to the incident polarization vector (i.e., an “×”-like pattern). The lobes of the cross-linear image form along the regions that are at 45 deg angles with respect to the incident polarization vector (i.e., a “+”-like pattern). Interestingly, the co-linear intensity distributions along the perpendicular axis appear higher than along the parallel axis, resulting in a somewhat asymmetrical oblong image. This may arise from preferential linear polarization preservation through ~lateral scattering in the plane perpendicular to the incident linear polarization vector; further investigation is needed to better understand this phenomenon.

**Fig. 3:**
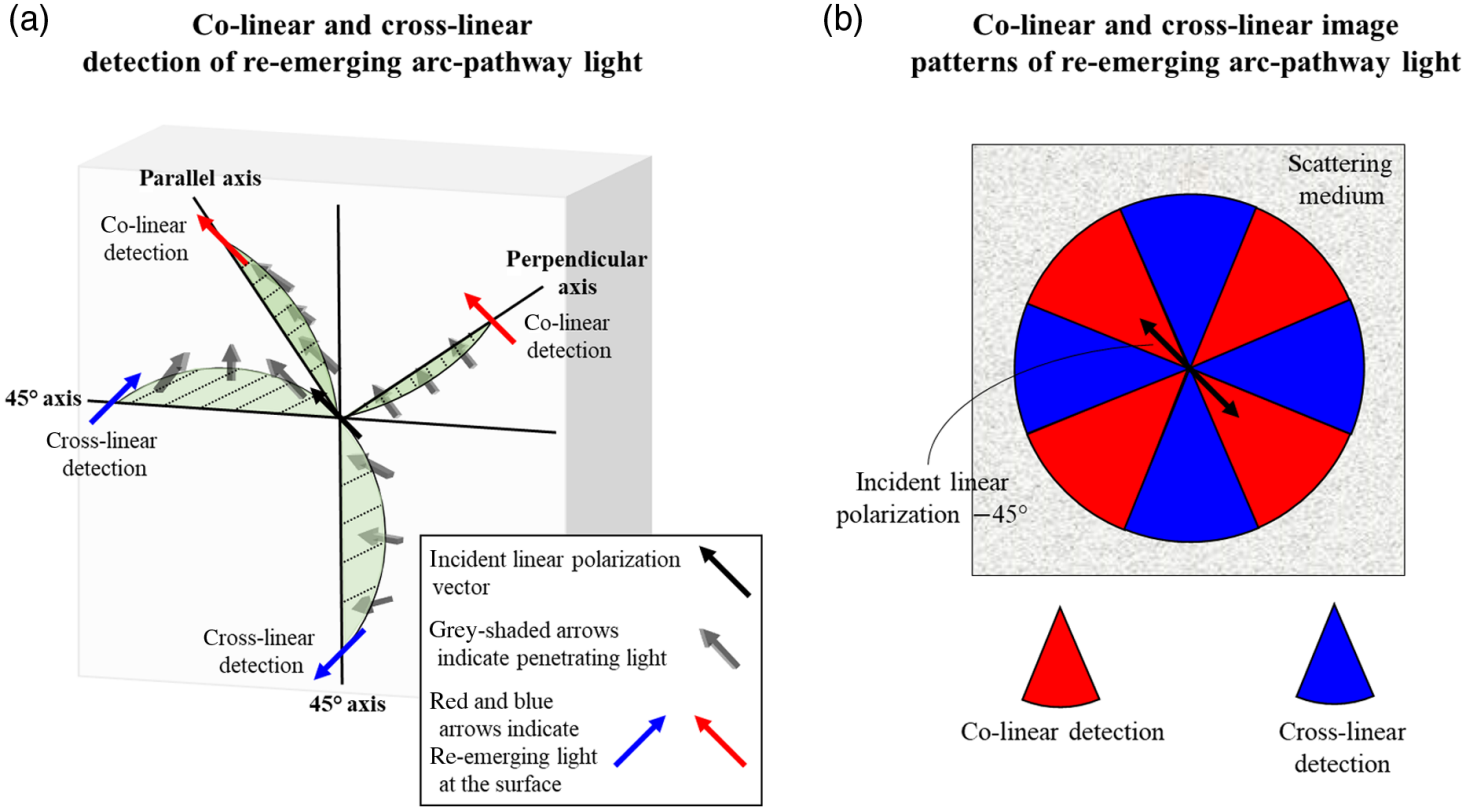
Geometrical effects on the linear polarization vector through penetrating arc-like pathways. (a) When linearly polarized light takes an arc-like pathway into the medium along the 45 deg axes, it re-emerges with a crossed orientation with respect to the incident polarization vector (colored blue). Conversely, when the light takes an arc-like pathway into the medium along the parallel or perpendicular axes, the polarization vector re-emerges with its orientation parallel to the incident polarization (colored red). See text for details. (b) Due to the geometric effects of arc-pathways, four-lobed patterns emerge from co- and cross-linear images whereby lobes form along perpendicular and parallel axes (red sectors) or at 45 deg (blue sectors) relative to the incident linear polarization vector, respectively.

The “+”-like pattern of the cross-linear image forms due to a multiple-scattering geometrical reorientation effect of penetrating arc-like pathways on the linear polarization orientation, as visualized in Fig. 3(a) (blue arrows) and Fig. 3(b) (blue sectors). Specifically, when the light takes on a forward-scattering arc-like pathway into the medium, the incident polarization vector can re-emerge with an orientation that is now perpendicular to the incident vector along the axes that are at 45 deg with respect to the incident linear polarization vector.^34^ In contrast, the “×”-like patterns form in co-linear images of Fig. 2(c) [and less clearly in Fig. 2(a)] since arc-like pathway light along the axes parallel and perpendicular to the incident polarization vector cannot become crossed due to the symmetry between the pathway direction and that vector. This is visualized by the red arrows in Fig. 3(a) and red sectors in Fig. 3(b).

Another feature of the co-linear images is the detection of the reflection-like orthogonalized light [see Fig. 1(b)] whose polarization orientation remains parallel to the incident linear polarization vector. [This is somewhat counterintuitive since orthogonalization implies that the scattered polarization vector becomes perpendicular to its incident state; however, this is due to the change of reference frame (propagation direction) whereby +45  deg polarization becomes −45  deg in the opposite direction of propagation, despite the physical polarization-axis orientation with respect to the optical table remaining unchanged.] Detection of the orthogonalized light gives rise to a very strong intensity near the illumination region of the co-linear image relative to the cross-linear image as shown in Fig. 2. It is mostly confined to the illumination region since in these interactions the medium essentially acts as a partial mirror at the incidence illumination spot.

Clearly, co- and cross-linear images, and the various spatial patterns within them, are dependent on sub-surface photon propagation pathway dynamics. The sampling depth effects of these linear polarization images can be further investigated through analysis of computational modeling statistics, which appear to be reliable as supported by the reasonable agreement between the simulation and experimental images.

**Fig. 4:**
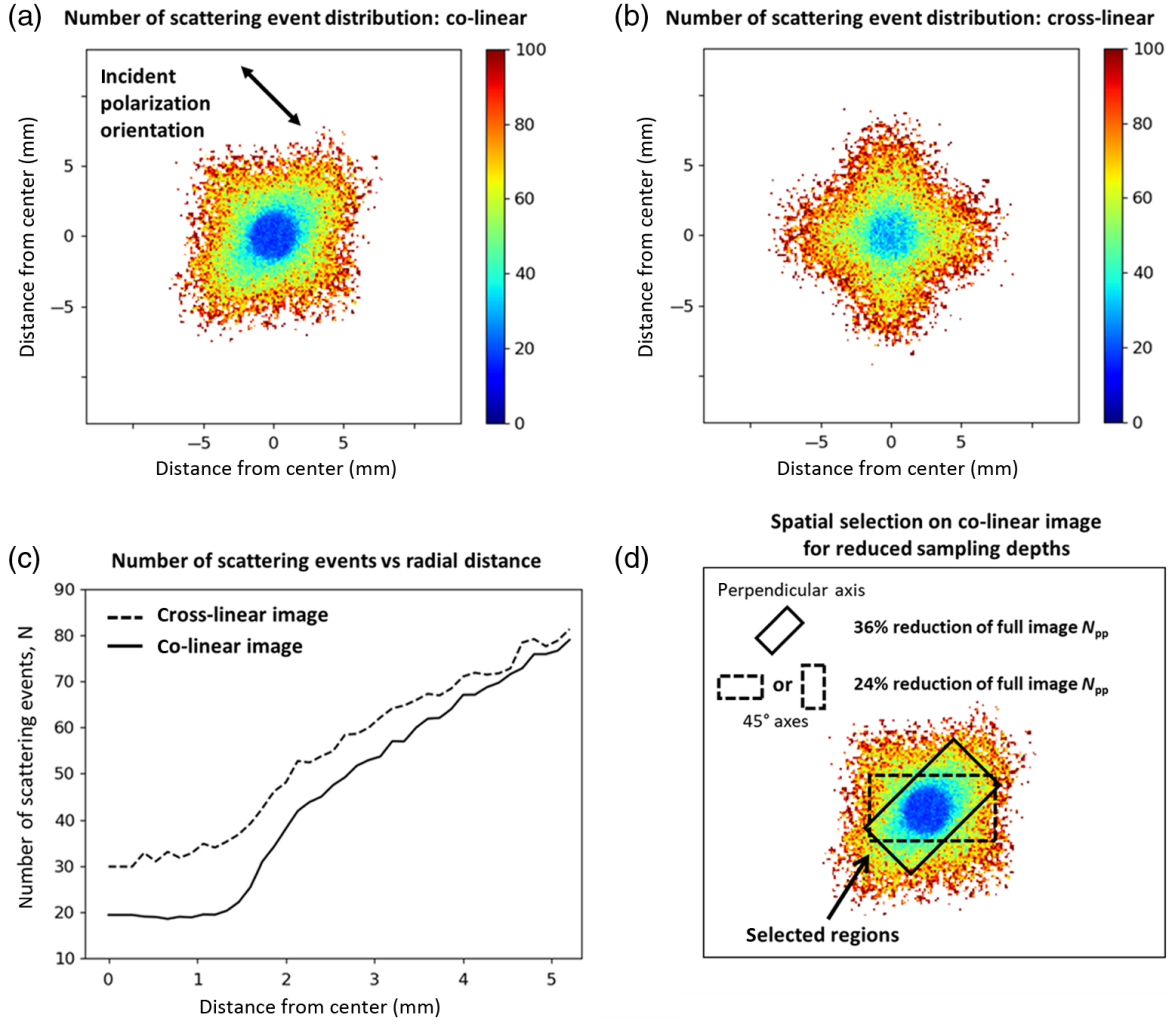
Monte Carlo analysis of the number of scattering events yields insight into spatially sensitive sampling depths. (a), (b) Distributions of the number of scattering events per pixel corresponding to the co-linear and cross-linear images in Figs. 2(c) and 2(d). (c) The radial profiles of the co-linear and cross-linear N distributions showing that the former offers the shallowest sampling (lowest N) closer to the illumination region. (d) Sampling depth can be further reduced by selecting pixels within specific regions of the co-linear image (in a). Selecting pixels within the solid-lined rectangle, oriented perpendicularly to the incident polarization vector, reduces the $N_{\mathrm{pp}}$ by 36% relative to that of the full image (see text for details on calculation of $N_{\mathrm{pp}}$), as compared to the more modest reduction in $N_{\mathrm{pp}}$ of 24% when selecting pixels within a rectangle oriented along the 45 deg axes (dashed outline).

Figure 4 shows the average number of scattering events N per detected co-linear [Fig. 4(a)] and [Fig. 4(b)] cross-linear photon packet at their corresponding spatial pixel locations. These are calculated by dividing the total number of scattering events at each pixel by the corresponding number of detected photons at each pixel [found in the intensity images of Figs. 2(c) and 2(d)]. It is evident that co-linear photons undergo less scattering, particularly in and close to the region of illumination. This is quantitatively shown by the radial profiles of the co- and cross-linear N-distributions in Fig. 4(c), whereby the largest difference in N is found at short radial distances; then both curves gradually converge with increasing radial distance until the profiles become ∼equal, indicating total depolarization. This reaffirms that co-linear polarization detection offers shallower sampling depths (as less scattering implies lower penetration depth).^3^

The oblong shape of the distribution in the co-linear image gives rise to a similarly shaped N-distribution, with regions along the “long” axis evidently encompassing most of the less-scattered light (low N values). For example, the light selected within a solid rectangle along that axis, such as the one drawn over the image in Fig. 4(d), appears to have undergone less scattering than if light was selected within a rectangle along any other axis. To test this hypothesis, we calculate the number of scattering events per photon per pixel ($N_{\mathrm{pp}}$) within that rectangle when it is oriented along the perpendicular axis (solid outline) and along the vertical or horizontal axis (i.e., the 45 deg axis; dashed outline). These $N_{\mathrm{pp}}$ values are calculated by summing the $N_{\text{per photon}}$ values at each pixel within the specified region, then dividing by the total number of pixels. As shown in Fig. 4(d), indeed the shallowest sampling depth is achieved through selection of light within the perpendicular axis rectangle, resulting in a 36% reduction of $N_{\mathrm{pp}}$ relative to the $N_{\mathrm{pp}}$ of the full co-linear image, as compared to the 24% reduction of $N_{\mathrm{pp}}$ when selecting light within the rectangle along the 45 deg axes.

It must be noted that one could instead simply select the light in the very sharply defined illumination region [see Figs. 2(c) and 4(a)] to enable the shallowest sampling depths; however, this sharp definition is a result of the flat-field beam used in the simulations (Gaussian beams are currently not supported). Since in practice most laser beams are Gaussian, experimental selection of the illumination region is not as trivial (no sharp cutoff) and the spatial selection discussed above is more practical. Finally, it should also be noted that the results presented in this section likely depends on the medium’s anisotropy parameter (g-factor)^50^ and possibly other optical properties; these will be explored in a future study.

Overall, it is shown that co-linear and cross-circular (helicity-flipped) detection are independent signal channels that are sensitive to reflection-like orthogonalized light. This type of light generally penetrates less deeply than the arc-like pathway light, which is much more prominent in cross-linear and co-circular detection configurations. In addition, certain regions within the co-linear and cross-circular images can be selected to further reduce the sampling depth, namely pixels closer to the illumination region (both images) and pixels along the perpendicular axis (co-linear image).

## 4 Polarimetric Spatial Field Selection: Differentiating Normal and Demineralized Teeth

This reduced-sampling-depth polarimetric approach can be applied to biological tissue to evaluate its potential biomedical utility. We thus employ normal and demineralized teeth as representative tissues that are known to have structurally different surface and sub-surface properties.^51^ Differentiation between these tooth types is an important clinical problem, as it can prevent cavities and tooth loss.^38^

**Fig. 5:**
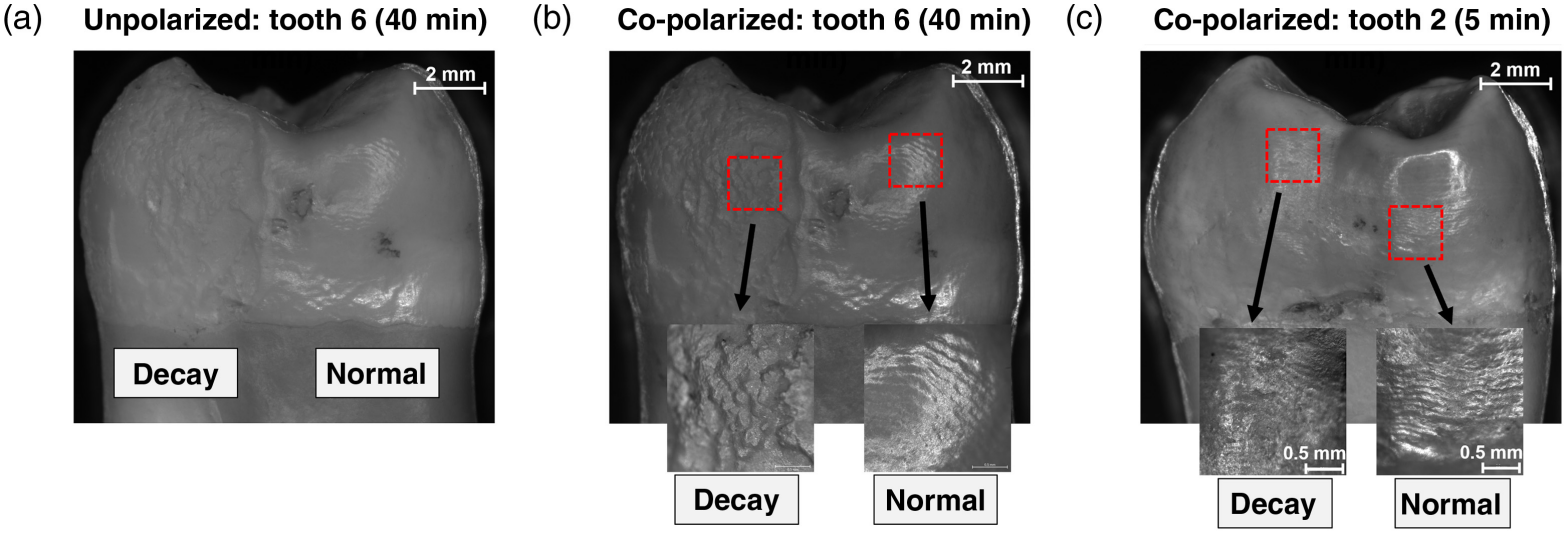
(a) Unpolarized and (b) co-linear polarized microscopic images of a tooth with a normal side and a 40-min acidified side (see labels) using a brightfield microscope (Zeiss AxioZoom.V16) at 8× magnification. Note that the co-polarized image accentuates the surface features. 63× magnified images of both sides of the tooth, shown as insets, exhibit a clear difference in the surface topology. (c) This difference becomes less apparent/indistinguishable for the tooth with a 5-min acidified side, even in the 63× magnified images (insets).

Polarimetric measurements were made on each tooth to differentiate the normal and acidified sides with a focus on superficial dental layers (enamel), and the differences between the two groups were quantified. Figures 5(a) and 5(b) show unpolarized and co-linear polarized images of a tooth with a normal side and a 40-min-acidified side at 8× magnification using a brightfield microscope (Zeiss AxioZoom.V16), respectively. 63× magnified co-polarized images of the normal and acidified sides are also shown as insets in Fig. 5(b) where a clear difference in surface topology is observed. Notice that the co-linear polarized image accentuates the surface features by minimizing the contributions of deeply penetrating (depolarized) light to improve contrast between the normal and acidified sides—a visual example of the heightened sensitivity to top dental layers gained by co-linear detection. However, shorter acidification times reduce such visual contrast; for example, the 5-min-acidified side of the tooth in Fig. 5(c) is difficult to discern, even at 63× magnification (the most prominent difference is likely its reduced glossiness). This highlights the challenge of early tooth decay detection and underscores the need for quantitative analysis.

The outermost layer of teeth, the enamel, consists primarily of enamel prisms surrounded by calcium hydroxyapatite.^37^^,^^38^ Early tooth decay typically involves demineralization of the enamel layer whereby bacteria-produced acids in the saliva dissolve the calcium hydroxyapatite.^37^ This leads to commonly observed increased surface roughness,^37^^,^^51^^–^^54^ as well as increased scattering (often quantified by the scattering coefficient) just below the surface.^53^^,^^55^^–^^58^ Since specular reflectance decreases with surface roughness,^59^ it is expected that orthogonalized light (i.e., specularly reflected light) intensity will be lower for demineralized teeth. Indeed, reduced specular reflectance has previously been observed for demineralized enamel suggesting its potential use for early tooth decay detection,^54^ although this study did not consider polarization properties. Furthermore, greater scattering coefficients typically result in higher depolarization.^47^^,^^60^^,^^61^ Thus, it is expected that the backscattered co-linear and cross-circular intensities from demineralized teeth will be lower relative to normal teeth. We explore this conjecture and its further refinement via judicious spatial selection of specific regions in these backscattered polarization images (as per methodology developed in the phantom study).

**Fig. 6:**
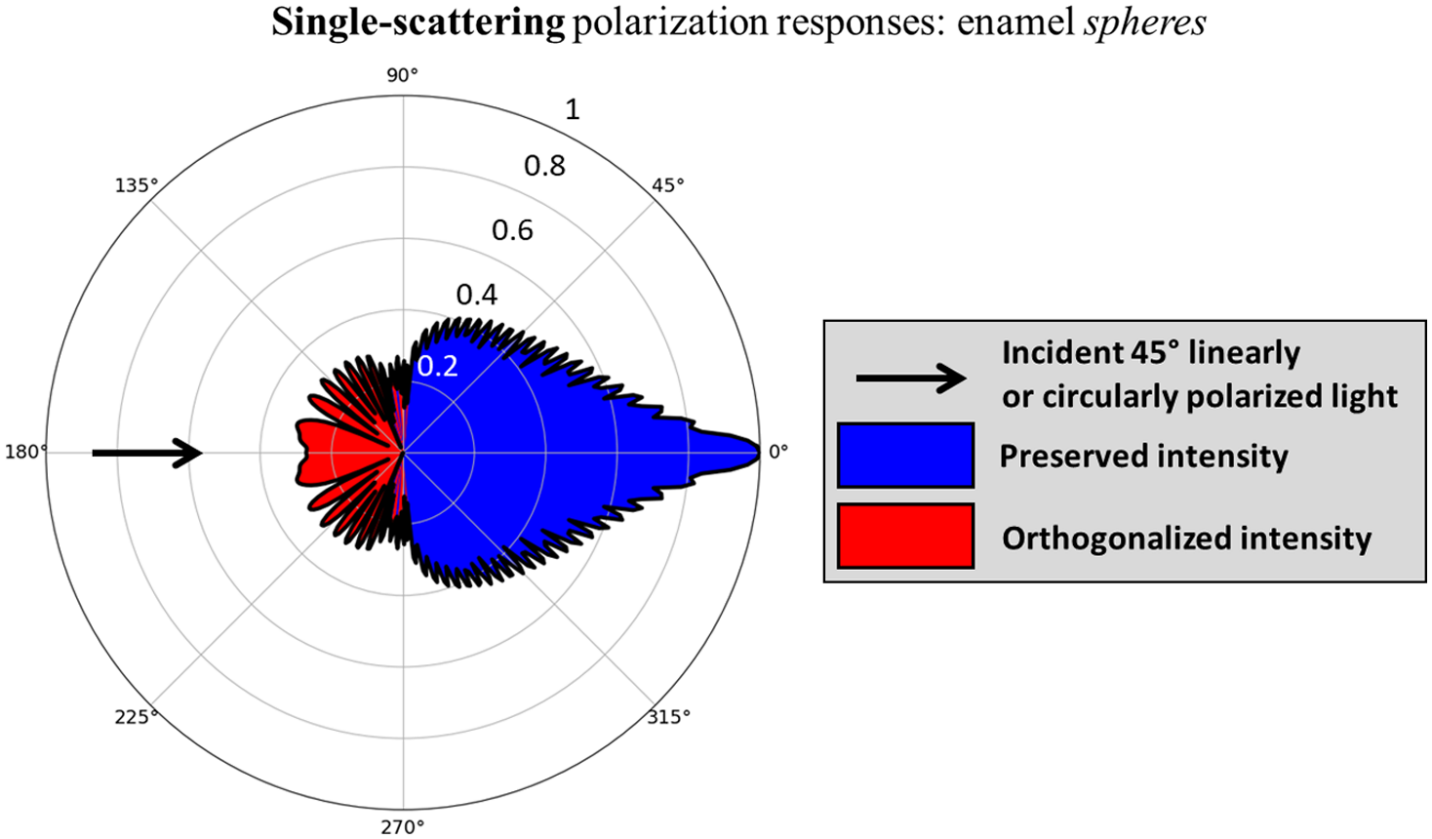
Polar plot showing the scattered 45 deg linear and circular polarization intensities from incident 45 deg linearly and circularly polarized light in dental scattering (enamel crystals in hydroxyapatite background). Orthogonalization of the incident polarization state occurs in the backward-hemisphere (90 deg to 270 deg), which likely results in shallower multiple-scattering pathways compared to preserved-polarization light (cf. Fig. 1).

Analogous to the phantom study, we may gain some theoretical insight into the orthogonalized light response from teeth from their single-scattering Mie optical properties. The thickness of the human enamel layer is estimated to be ∼1.4 to 1.7 mm.^62^^–^^65^ As average polarized light sampling depths in turbid biological tissues are typically estimated to be ∼2  mm,^15^^,^^16^^,^^66^^–^^68^ and likely much shallower for orthogonalized light, we will only consider the scattering dynamics in the enamel layer. Enamel prisms are thought to be dominant scatterers, surrounded by an interprismatic substance consisting of proteins and water.^69^ To first order, we can approximate the prisms as spheres,^70^ enabling direct use of Mie theory and Stokes-Mueller calculus to compute the single scattering angular distributions of linear and circular polarization intensity. The Mie input parameters for the calculation were: light wavelength in vacuum λ=543.5  nm, host medium refractive index $n_{m}=1.573$ (interprismatic substance^71^), scatterer refractive index $n_{s}=1.619$ (enamel prisms^71^), and scatterer representative linear dimension d=5.75  μm (prism diameters typically range from 4 to 7.5  μm^69^). Similar to the polar plot in Fig. 1, Fig. 6 shows that backward-scatter events result in orthogonalization and forward-scattering results in preservation of polarization; the details of the resulting distribution shape are not important due to the uncertainly in the dental properties and calculation assumptions. But overall, we note that orthogonalized light will likely penetrate less deeply [e.g., as schematized in Fig. 1(b)] and is detectable through cross-circular and co-linear polarimetric arrangements.

**Fig. 7:**
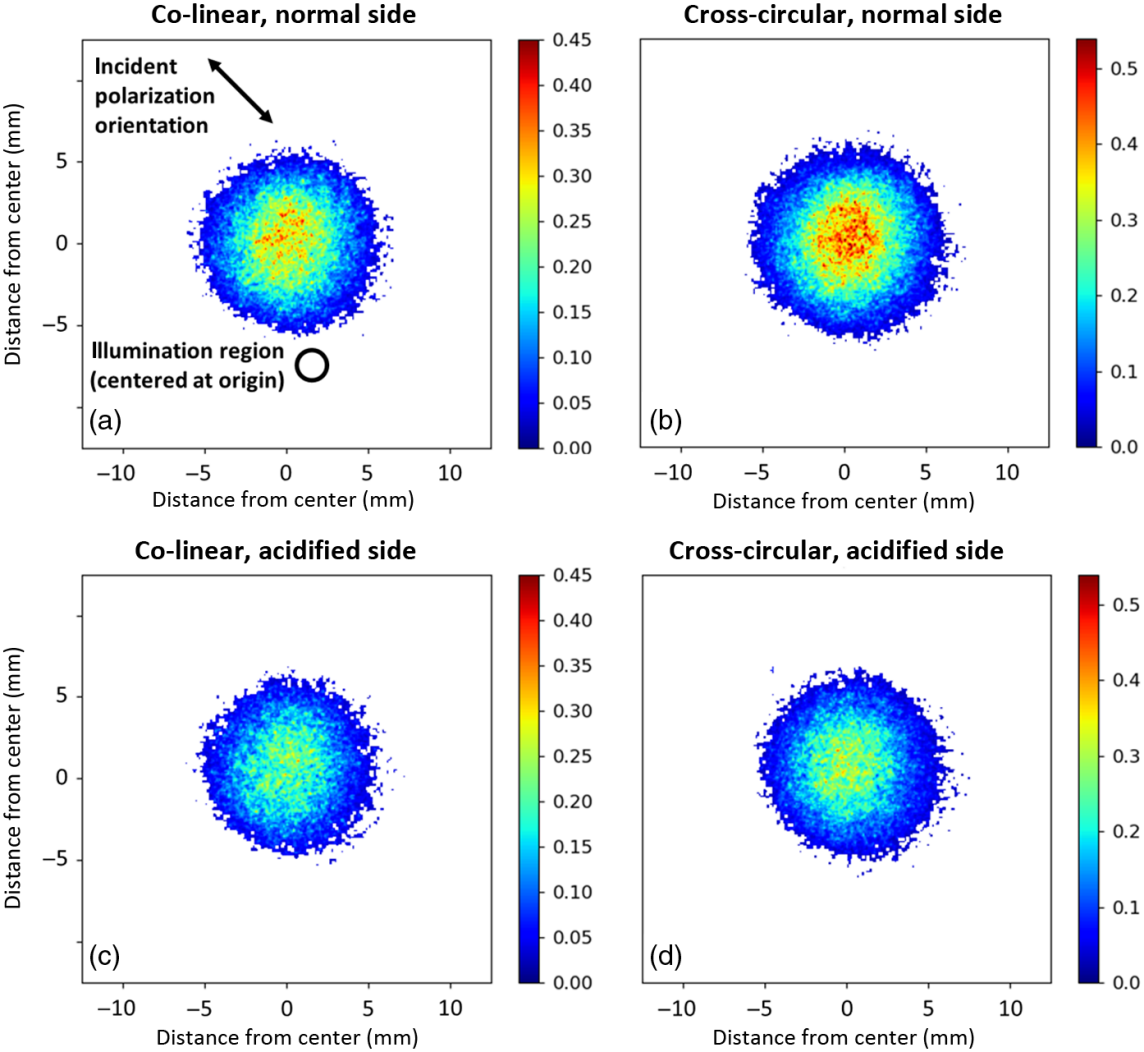
(a), (c) Co-linear and (b), (d) cross-circular images of the normal and the 40-min-acidified side of a tooth [same sample as shown in shown in Figs. 5(a) and 5(b)]. There is a clear decrease in the polarization intensities corresponding to the acidified side, attributable to the roughened surface and more depolarizing sub-surface scattering. No spatial patterns are visually apparent in the co-linear images (see text for details).

Figures 7(a) and 7(c) show co-linear and Figs. 7(b) and 7(d) shows cross-circular images of the normal and the 40-min-acidified sides of a tooth [same as shown in Figs. 5(a) and 5(b)]. The demineralized side results in noticeably weaker intensities (bottom row compared to top row). However, there is no obvious visibly apparent “×”-like four-lobed pattern in the co-linear images such as those seen in the phantom case of Fig. 2. This may be due to the higher heterogeneity of teeth and broader refractive index and scatterer size distributions compared with the microsphere suspension, yielding a larger depolarized background with more isotropic intensity distributions; however, this is not yet fully understood. There does appear to be somewhat of a higher intensity along the perpendicular axis of many of the co-linear images, for example, in Fig. 7(a), but this is difficult to ascertain qualitatively. Thus, we turn to the quantitative analysis of the spatial-selection methodology developed in the earlier phantom study to quantify polarimetric enamel changes even in this challenging case of visually unobservable patterns.

**Fig. 8:**
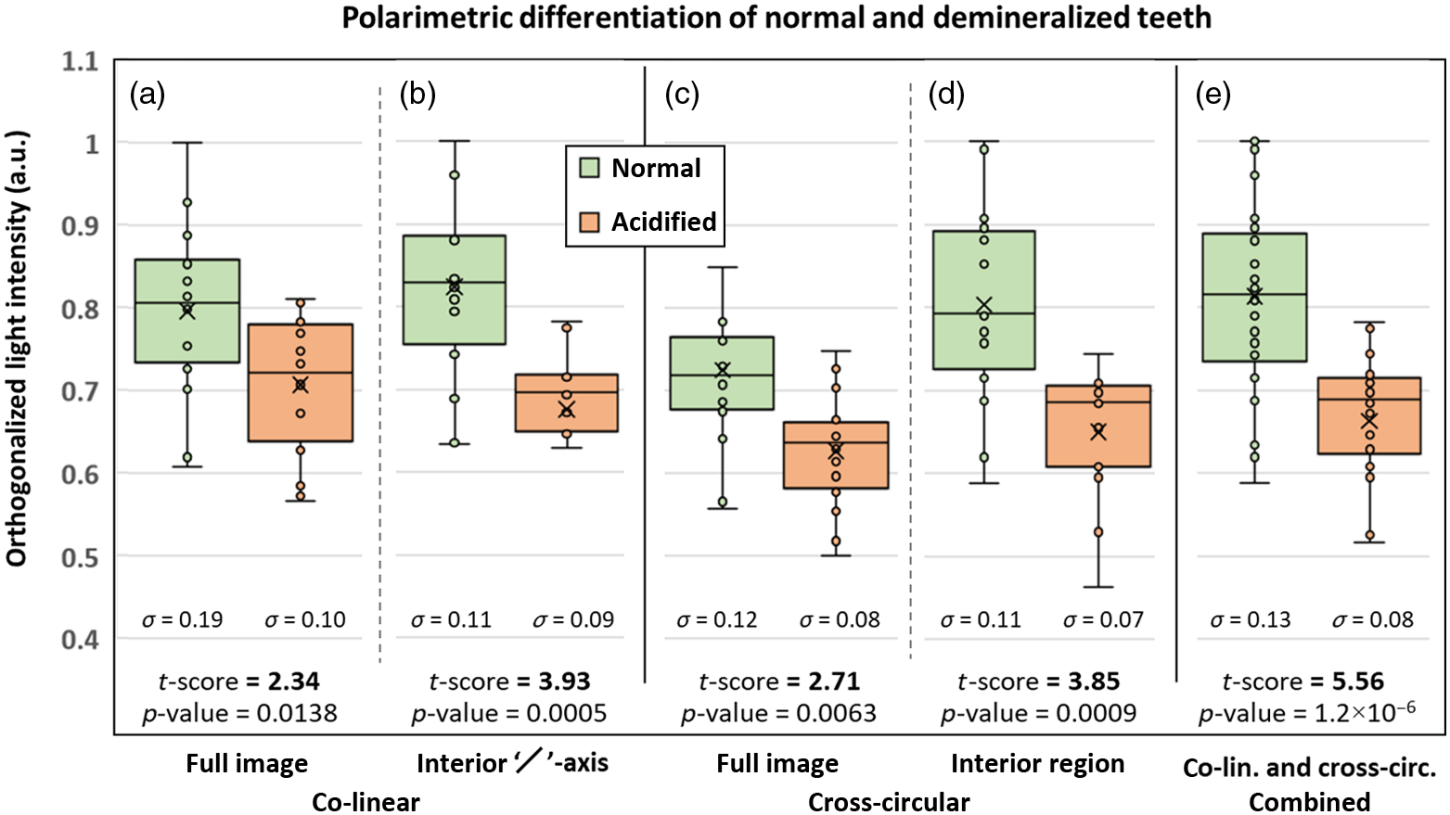
Box-and-whisker plots of the intensity metrics corresponding to the normal and acidified sides of the teeth. The individual data points are also shown, as well as the mean values (indicated by the “×” symbols) and standard deviations (denoted by the “σ” symbols). Five metrics were used to generate five pairs of box-and-whisker plots: summed pixel intensities from (a) the co-linear full image region, (b) co-linear interior perpendicular axis region (denoted by the “/” symbol), (c) cross-circular full image region, and (d) cross-circular interior region. The “interior region” metrics yield the highest separations. (e) The box plot when the data points from both (b) and (d) are included, yielding the highest t-score via the doubling of the sample size.

To test if co-linear and cross-circular intensities are lower in demineralized teeth, the pixel values from the normal and acidified sides of each tooth were summed over the full image and compared using t-score values. The t-scores are simple and effective measures of relative difference between the two groups (e.g., intensities from normal and acidified sides),^72^ calculated as

$$t\text{-score}=\frac{\left({\overset{¯}{x}}_{1}-{\overset{¯}{x}}_{2}\right)}{\sqrt{S^{2}(\frac{1}{n_{1}}+\frac{1}{n_{2}})}},$$ (3)

where ${\overset{¯}{x}}_{1}$ and ${\overset{¯}{x}}_{2}$ are the means of the normal and acidified tooth sides, respectively, $n_{1}$ and $n_{2}$ are the number of data points corresponding to the normal and acidified images, and S is the standard deviation of all the data points. Indeed, the co-linear and cross-circular full image intensities are lower for the demineralized teeth, as shown in the box plots of Figs. 8(a) and 8(c), respectively, yielding t-scores of 2.34 (co-linear) and 2.71 (cross-circular) with statistically significant separation (p-values<0.05). The lower intensities are attributed mainly to the roughened surfaces and somewhat to the more depolarizing sub-surfaces.

The spatial selection methods developed in the phantom investigation can be applied to improve this differentiation. Specifically, for circularly polarized light, the pixels within the illumination region are selected for summation, and for linearly polarized light, the pixels along the perpendicular axis within the illumination region are selected for summation. The illumination region encompasses the central pixels within a 3 mm diameter (i.e., the incident beam diameter). The width of the perpendicular axis rectangle is taken as 1/2 of the illumination region diameter (1.5 mm). These dimensions were chosen to best capture light along the perpendicular axis direction while avoiding “crosstalk” from the higher-order scattering along the 45 deg axes’ directions; again, the “optimal” dimensions of this region should be further investigated. As shown in Fig. 8, the intensities of the co-linear perpendicular axis within the interior region [Fig. 8(b)] and the cross-circular interior regions [Fig. 8(d)] yield considerably greater differentiation between normal and demineralized teeth, with t-scores of 3.93 and 3.85, respectively. Indeed, it appears that the polarimetric spatial field selection approach enhances sensitivity to the superficial regions of the teeth where they are structurally most different. Finally, combining both the cross-linear and co-linear measurements of Figs. 8(b) and 8(d) yields more robust statistical differentiation [Fig. 8(e)] in part due to effectively doubling the sample size (more measurements improve the signal-to-noise ratio). More advanced ways of combining the linear and circular data (e.g., product, ratio, weighted average, etc.) could further improve differentiation and should be investigated.

**Fig. 9:**
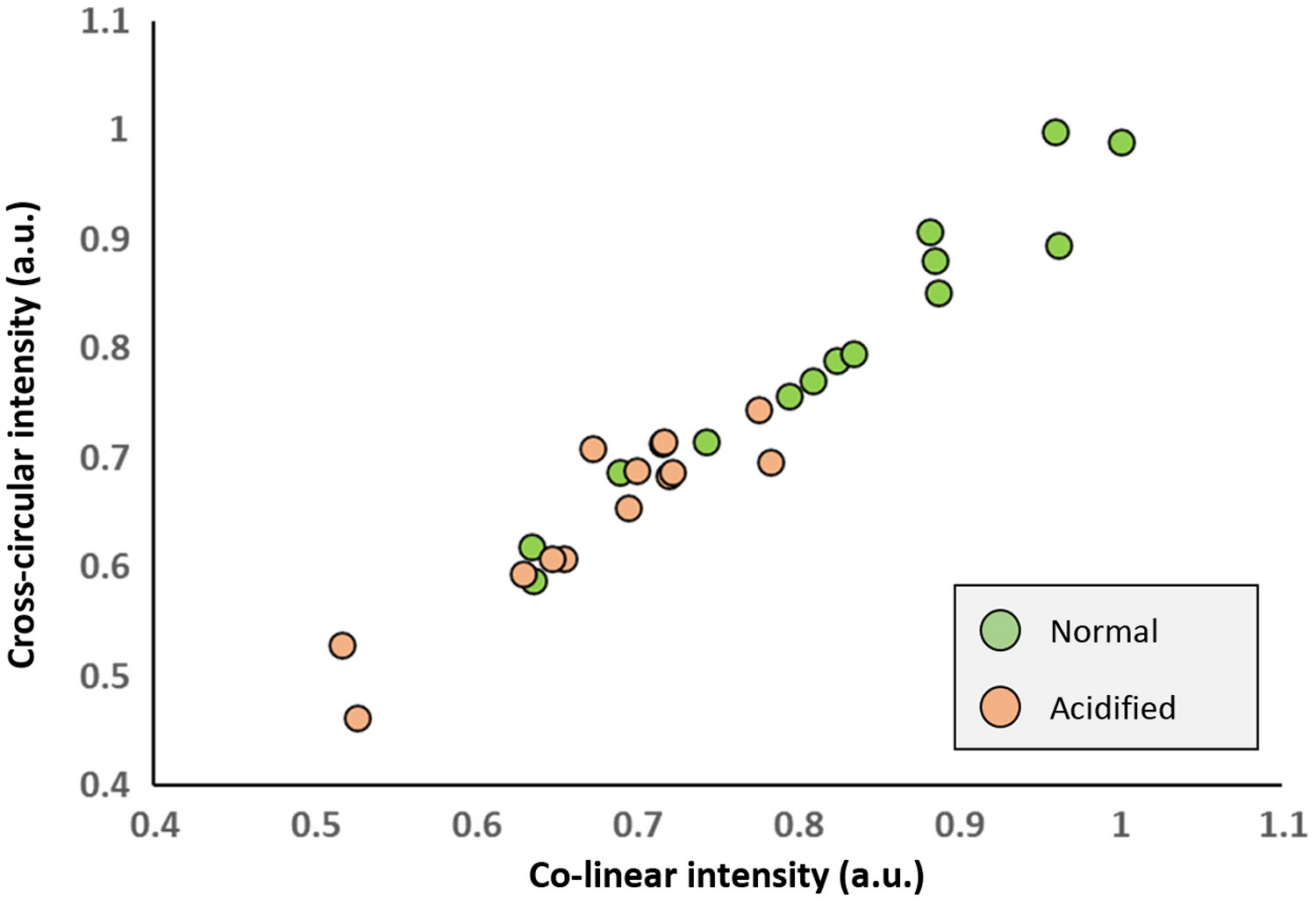
Polarimetric mapping of cross-circular and co-linear intensity value pairs shows separation of most normal and acidified data points. This may be useful for classification of these tooth types or potential grading of decay based on datapoint positions (e.g., lower x, y values indicate severe decay).

Another potential method of results analysis is to pair the data points as (x,y) = (co-linear intensity, cross-circular intensity) from each measured tooth spot to form a “polarimetric map” plot as shown in Fig. 9. The normal-tooth points are mostly separated from the acidified-tooth points. Such a map may be useful in classifying teeth as healthy or decayed upon co-linear and cross-circular measurement. Additional parameters can be incorporated to improve classification accuracy, such as speckle-based metrics, which have shown promise in tooth decay detection^73^^–^^75^ and can be measured in a polarimetric setup.^76^^,^^77^ Finally, it appears that the points form a somewhat straight line and their positions may be dependent on surface roughness (e.g., smoother surfaces yield higher x,y values), possibly enabling a calibration line to grade severity of tooth decay.

## 5 Conclusion

Here, it is demonstrated that optical sensitivity to the superficial regions of tissue phantoms and normal/demineralized teeth can be enhanced through a combination of co-linear and cross-circular polarization detection along with judicious spatial selection of specific regions in the backscattered images. In the circular case, the field closer to the illumination region can be selected to reduce the sampling depth. In the linear case, the axis that is perpendicular to the incident linear polarization vector within an area closer to the illumination region can be selected. These results are supported by (1) experimental and computational modeling evidence using polystyrene microsphere suspensions, and (2) experimental polarimetric measurements on normal and demineralized teeth, which are known to differ in their surface/sub-surface structures.

In the future, larger tooth sample sizes should be used to further evaluate the approach for this important clinical problem. This dual-channel polarimetric spatial field selection approach can be combined with other depth-sensitive techniques, such as angular-resolved detection,^40^ elliptical polarization measurements,^13^^–^^16^ and multispectral approaches,^21^ for increased control over sampling depths. This may also prove useful for detecting cell nuclear pleomorphism—a hallmark of cancer^78^—during malignant progression in epithelial tissues, where the vast majority of cancers develop (80% to 90%).^79^^,^^80^ These tissues are mostly cellular and line body surfaces and organs;^81^ it is important to maximize signals from these relatively thin superficial linings while minimizing light penetration beyond the epithelial layer—similar to the tooth decay detection problem. Indeed, a powerful polarimetric technique may be formed, which combines this added cellular probing capability to the well-demonstrated collagen sensing via polarization retardance imaging.^82^^,^^83^

## Code, Data, and Materials Availability

The datasets used for this study are not publicly available at this time but may be obtained upon reasonable request.
